# Association of Rumination Time with Metabolic Imbalance and Milk Quality Traits in Holstein Cattle

**DOI:** 10.3390/biology15070581

**Published:** 2026-04-05

**Authors:** Samanta Grigė, Akvilė Girdauskaitė, Lina Anskienė, Inga Sabeckienė, Karina Džermeikaitė, Justina Krištolaitytė, Dovilė Malašauskienė, Mindaugas Televičius, Ramūnas Antanaitis

**Affiliations:** 1 Large Animal Clinic, Veterinary Academy, Lithuanian University of Health Sciences, LT-47181 Kaunas, Lithuania; akvile.girdauskaite@lsmu.lt (A.G.); inga.sabeckiene@lsmu.lt (I.S.); karina.dzermeikaite@lsmu.lt (K.D.); justina.kristolaityte@lsmu.lt (J.K.); dovile.malasauskiene@lsmu.lt (D.M.); mindaugas.televicius@lsmu.lt (M.T.); ramunas.antanaitis@lsmu.lt (R.A.); 2 Department of Animal Breeding, Faculty of Animal Sciences, Lithuanian University of Health Sciences, LT-47181 Kaunas, Lithuania; lina.anskiene@lsmu.lt

**Keywords:** Innovative technologies, biomarkers, milk composition, rumination

## Abstract

Rumination time is considered a sensitive behavioral indicator of physiological and metabolic status in dairy cows, yet its relationships with biochemical and milk quality parameters under commercial robotic milking conditions remain insufficiently described. This study combined precision monitoring technologies, serum biochemical profiling, and in-line milk analysis to evaluate physiological differences among early-lactation Holstein cows according to rumination time. A total of 88 cows were classified into three rumination time categories (>527, 412–527, and <412 min/day). Milk production traits, milk quality indicators, and blood biochemical parameters were compared among groups, and univariable regression analysis was performed to identify variables associated with rumination time. Cows in the low rumination group showed higher milk temperature, electrical conductivity, and somatic cell count, as well as lower milk protein percentage. They also showed higher concentrations of total protein, urea, gamma-glutamyl transferase, and lactate dehydrogenase, while triglyceride concentrations were lower. Regression analysis identified electrical milk conductivity, creatinine, magnesium, potassium, and chloride as variables associated with rumination time. These findings indicate that reduced rumination time is associated with changes in milk quality and biochemical parameters in early-lactation dairy cows, suggesting that rumination monitoring may provide useful information for identifying cows experiencing physiological and metabolic challenges under commercial farming conditions.

## Simple Summary

Monitoring rumination, which is the time cows spend chewing cud, is an important way to evaluate the health and well-being of dairy cows, especially in early lactation when cows experience high metabolic stress. Modern robotic milking systems allow continuous recording of rumination behavior, milk production, and milk quality traits, which may help identify cows experiencing health or metabolic problems at an early stage. In this study, we compared milk production, milk quality, and blood biochemical parameters in early-lactation dairy cows with different rumination times under commercial farm conditions. Cows with lower rumination time showed higher milk electrical conductivity, higher somatic cell count, and changes in several blood biochemical parameters related to protein metabolism, enzyme activity, and energy metabolism. These results suggest that reduced rumination time may be associated with physiological and metabolic changes during early lactation. Combining rumination monitoring with milk and blood indicators may help farmers and veterinarians identify cows that require closer monitoring and management during early lactation. This approach may contribute to improved animal health, welfare, and farm management.

## 1. Introduction

In dairy cattle, rumination is a major behavioral activity that is essential to the regulation of feed intake and rumen function. Rumination time (RT) in cows usually ranges between 400 and 600 min per day, depending on diet, lactation stage, and individual variation, according to more accurate measurements of rumination made possible by modern automated behavior-monitoring systems [1,2]. Changes in RT are becoming more widely acknowledged as sensitive markers of metabolic strain during the transition phase because rumination reacts quickly to decreases in dry matter intake and changes in the rumen environment [1,3].

A growing body of research shows that declines in RT often precede metabolic disorders, particularly subclinical ketosis. Several field studies have reported that cows developing subclinical ketosis show a detectable decline in RT two to four days before increases in blood β-hydroxybutyrate concentrations are observed [4,5]. This may be explained by reduced feed intake and decreased rumen fill during the early stages of negative energy balance, which lead to reduced rumination activity before measurable changes appear in blood metabolic markers [6]. However, the strength and timing of this relationship may vary depending on diet, management system, and stage of lactation. Reduced RT during the peripartum period has also been associated with an increased risk of metritis and systemic inflammatory responses, further supporting the role of rumination behavior as a non-invasive indicator associated with health status [7].

The development of precision livestock farming technologies has greatly increased the availability of continuous behavioral data and improved the ability to monitor dairy cow health and welfare under commercial conditions [8]. Neck-mounted rumination sensors have been validated in different housing systems and generally show good agreement with direct observation, making them suitable for large-scale and real-time monitoring [9,10]. However, some limitations should be considered, including variability between devices and algorithms, potential effects of collar position and cow activity on sensor accuracy, and challenges in interpreting behavioral data without additional physiological or production information. Therefore, rumination data are most useful when interpreted together with milk production, milk quality, and biochemical indicators.

Automatic milking systems with in-line analyzers simultaneously produce high-frequency data on milk composition, including fat, protein, lactose, electrical conductivity, and somatic cell count [11]. Since changes in electrical conductivity may indicate early mastitis and changes in lactose concentration or the fat-to-protein ratio sometimes indicate systemic inflammation or a negative energy balance, these milk-derived measurements offer instant insight into udder health and metabolic status [12]. When somatic cell count increases or when inflammatory events compromise mammary epithelial function, milk lactose concentration decreases, making it very informative [13]. Similarly, high values of the milk fat-to-protein ratio (FPR) indicate enhanced adipose mobilization and a higher risk of ketosis in the early stages of lactation, and it has been extensively confirmed as a useful indicator of negative energy balance [14]. These in-line milk characteristics offer a supplementary physiological perspective when combined with behavioral records like RT, providing a more thorough and trustworthy evaluation of cow health under commercial dairy farm conditions and providing useful context for interpreting changes in rumination behavior [15].

Despite these advancements, comparatively few studies have assessed rumination behavior in early-lactation cows under commercial robotic milking circumstances in conjunction with both milk content and serum biochemical markers. By combining these complementary data streams, it may be possible to identify cows experiencing inflammatory or metabolic stress more accurately and to help build more sophisticated monitoring tools for herd management.

Therefore, the aim of this study was to integrate rumination sensor data, AMS-derived milk composition traits and serum biochemical indicators to characterize physiological differences among early-lactation Holstein cows with varying daily RT under commercial farm conditions. We hypothesized that cows with lower rumination time would exhibit biochemical and milk composition changes associated with metabolic stress, altered liver function indicators, and poorer udder health compared with cows with higher rumination time.

## 2. Materials and Methods

The animal procedures were reviewed and authorized by the State Food and Veterinary Service of the Republic of Lithuania (permit No. 135834789). All work complied with national regulations and institutional guidelines governing the use of animals for research. The field component of the study took place in September 2025 on a commercial dairy unit situated in Northern Europe.

Cows were fed twice daily (07:00–08:00 and 15:00–16:00). Automatic feed pushers redistributed the ration several times daily to maintain feed availability. Rations were formulated by a certified dairy nutrition specialist in line with NRC nutrient recommendations to match the cows’ physiological demands. The lactating cows were fed a total mixed ration (TMR) formulated to meet the nutritional requirements of a 550 kg Holstein cow producing approximately 35 kg of milk per day. The ration contained 48.8% dry matter (DM) and provided 1.6 Mcal NEL/kg DM. The chemical composition of the diet on a DM basis was as follows: neutral detergent fiber 28.2%, acid detergent fiber (ADF) 19.8%, non-fiber carbohydrates 38.7%, and crude protein 15.8%. Grain-based concentrate represented approximately 50% of the dietary DM. The forage portion consisted primarily of maize silage (approximately 30% of DM), supplemented with grass silage (approximately 10%), grass hay (approximately 4%), and smaller amounts of wheat straw, lucerne hay (13% crude protein), and sugar beet pulp silage. A commercial compound feed was included according to the farm’s standard feeding strategy. The TMR was prepared fresh daily and offered ad libitum, and cows had free access to clean drinking water.

Cows were housed in a loose-housing system and fed the same TMR throughout the year. The average annual energy-corrected milk yield per cow in 2024 ranged from 10,300 to 11,900 kg, with mean milk protein and fat contents of 3.4–3.6% and 4.1–4.2%, respectively.

### 2.1. Data Collection

Milking was carried out using Lely Astronaut^®^ A3 robotic systems (Lely, Maassluis, The Netherlands) operating under free-cow traffic. To promote voluntary entry, each cow received approximately 2 kg of concentrate per day within the milking unit. The robots continuously recorded milk production traits—milk temperature (T), milk yield (MY), milk fat (MF), milk protein (MP), lactose, somatic cell count (SCC), fat-to-protein ratio (FPR), and milk electrical conductivity—which was measured automatically by the Lely Astronaut system at each milking. The system reports conductivity as a proprietary Lely conductivity score (dimensionless), calculated from quarter-level measurements; the mean value of the four quarters was used for statistical analysis. All data were stored automatically in the Lely T4C herd management software (version 3.12). For analytical purposes, daily averaged in-line measurements corresponding to each cow’s blood sampling date were extracted to ensure synchronization with serum biochemical values.

Rumination-related information was recorded using Lely Qwes-H/HR neck-mounted tags (Lely, Maassluis, The Netherlands). These units contain accelerometers and use jaw movement pattern recognition to classify rumination activity. Data were logged at two-minute intervals and transmitted wirelessly to the barn antennae, where they were integrated into the T4C system. The algorithm and hardware are widely used in commercial herds and have been validated for monitoring rumination behavior [15].

Blood samples were collected at 10:00 a.m., before the afternoon feeding. Approximately 10 mL of blood was drawn from the coccygeal vein into plain evacuated tubes (BD Vacutainer, Crawley, UK). Blood samples were allowed to clot at room temperature and were subsequently stored at 4 °C. Within 2 h of collection, the samples were transported to the Laboratory of Clinical Tests, Large Animal Clinic, Veterinary Academy, Lithuanian University of Health Sciences for analysis. After centrifugation, serum was separated and analyzed immediately upon arrival at the laboratory. All samples were analyzed in a single analytical batch. Calibration and internal quality control procedures were performed according to the manufacturers’ recommendations.

Serum biochemistry was performed using a Hitachi 705 analyzer (Hitachi, Tokyo, Japan) with DiaSys reagent kits (Diagnostic Systems GmbH, Düsseldorf, Germany). Non-esterified fatty acids (NEFAs) were quantified with an Rx Daytona automated analyzer (Randox Laboratories Ltd., London, UK). Calibration procedures and internal quality-control checks followed the manufacturers’ recommendations. The Hitachi 705 was calibrated using DiaSys multipoint standards incorporating reagent-lot-specific factors.

The following biochemical parameters were assessed: albumin (ALB), aspartate aminotransferase (AST), gamma-glutamyltransferase (GGT), alanine aminotransferase (ALT), calcium (Ca), creatinine (CREA), C-reactive protein (CRP), iron (Fe), glucose (GLUC), lactate dehydrogenase (LDH), magnesium (Mg), phosphorus (PHOS), total protein (TP), triglycerides (TRIG), urea (UREA), sodium (Na), potassium (K) and chloride (Cl).

From a herd comprising approximately 2300 Holstein cows, a subset of early-lactation cows was selected for inclusion in this observational study. Cows between 2 and 100 days in milk were included to represent the early-lactation period, which includes the transition period and the subsequent phase of metabolic adaptation. Animals were selected from the commercial herd based on availability and data completeness from the automated milking and rumination monitoring systems, and only clinically healthy cows were included. All cows underwent a standardized clinical examination performed by a veterinarian prior to inclusion. The examination included assessment of general health status, rectal temperature, heart rate, respiratory rate, rumen motility, locomotion, udder health, and evaluation for signs of metritis, displaced abomasum, or gastrointestinal disorders. Cows showing signs of clinical disease, such as clinical mastitis, lameness, metritis, displaced abomasum, or gastrointestinal disturbances, were excluded. Subclinical mastitis and subclinical metabolic disorders were not used as exclusion criteria and were evaluated later using somatic cell count data and blood biochemical parameters as part of the study analysis. Cows were also excluded if behavioral, production, or health records were incomplete. Each cow was sampled once for blood analysis. A total of 88 cows met the inclusion criteria and were included in the final dataset (Table 1). After data collection, cows were retrospectively classified into RT categories based on daily rumination duration using previously published ranges reported by Mikuła et al. [16], who categorized cows according to daily rumination time. However, because rumination time is influenced by diet, lactation stage, housing, and management conditions, these thresholds should be interpreted as practical grouping criteria rather than fixed biological cut-off values. In the present study, the thresholds were used to classify cows into relatively higher, medium, and lower rumination groups within the study population to facilitate comparison of physiological, biochemical, and milk composition parameters among cows with different levels of rumination activity:

Group 1: Cows exceeding 527 min of rumination per day (*n* = 31).

Group 2: Cows ruminating between 412 and 527 min per day (*n* = 34).

Group 3: Cows ruminating less than 412 min per day (*n* = 23).

Milk production and sensorderived data from the Lely robotic milking system were extracted for each cow on the same day that the blood sample was obtained.

**Table 1:** Descriptive characteristics of the study cows for the study population and according to rumination time groups.

Variable	Study Population (Mean ± SD)	Group 1	Group 2	Group 3
Days in milk (DIM)	46.3 ± 25.4	48.1 ± 26.2	45.7 ± 24.8	44.9 ± 25.1
Milk yield (MY) (kg/day)	35.9 ± 15.7	39.2 ± 11.8	34.2 ± 14.2	39.4 ± 30.7
Lactation number	2.6 ± 1.4	2.5 ± 1.3	2.4 ± 1.2	2.9 ± 1.5

Descriptive characteristics of the study cows for the study population and according to rumination time groups. Group 1: cows with rumination time > 527 min/day; Group 2: cows with rumination time 412–527 min/day; Group 3: cows with rumination time < 412 min/day. DIM—days in milk; SD—standard deviation. Data are presented as mean ± SD.

### 2.2. Statistical Analysis

Statistical analyses were performed using IBM SPSS Statistics version 25 (IBM Corp., Armonk, NY, USA). Prior to analysis, data distribution was assessed using the Shapiro–Wilk test. Variables that were not normal were log-transformed prior to analysis to achieve an approximate normal distribution. SCC was log_2_-transformed prior to analysis. Descriptive statistics are presented as mean ± standard deviation (SD). Differences among the three RT groups were evaluated using one-way analysis of variance (ANOVA). Days in milk and lactation number were evaluated as potential confounders and were compared among groups descriptively. When a significant overall effect was detected, Tukey’s post hoc test was used for pairwise comparisons between groups. In addition, linear regression analysis was performed to evaluate the association between rumination time and selected milk composition and blood biochemical parameters. In addition, univariable linear regression analysis was performed to evaluate the association between rumination time (dependent variable) and individual milk composition and blood biochemical parameters (independent variables). Each predictor was entered into the model separately. Regression coefficients (B), 95% confidence intervals (CI), and *p*-values were calculated for each model. Because the aim of the regression analysis was exploratory, multivariable model building and collinearity diagnostics were not performed.

## 3. Results

### 3.1. Milk Composition and Milk Quality Traits

Mean milk temperature differed among groups (Table 2). Group 3 had the highest mean T, which was 1.28% higher than in Group 1 and 0.59% higher than in Group 2. The difference between Groups 1 and 3 was significant (*p* < 0.05), whereas differences between Groups 1 and 2 and between Groups 2 and 3 were not significant (*p* > 0.05).

ECM differed among groups (*p* < 0.05), although pairwise comparisons did not reveal significant differences between individual groups. Somatic cell count (log_2_ SCC) differed significantly among groups (*p* < 0.001), with higher values observed in Group 3 compared with Groups 1 and 2, while Groups 1 and 2 did not differ significantly.

Milk protein percentage differed among groups (*p* < 0.05), with the lowest values observed in Group 1 and the highest in Group 3. Pairwise comparisons indicated significant differences between Groups 1 and 3 and between Groups 2 and 3, whereas Groups 1 and 2 did not differ significantly. Milk fat, yield, and lactose concentration did not differ significantly among rumination groups (*p* > 0.05) (Table 3).

### 3.2. Blood Biochemical Parameters

Blood biochemical parameters across rumination groups are presented in Table 2 and Table 3. Serum ALB, Ca, Fe, PHOS, Na, K, Cl, CREA, AST, and NEFA concentrations did not differ significantly among rumination groups (*p* > 0.05).

CRP concentration differed significantly among groups (*p* < 0.01), with higher values observed in Group 1 compared with Groups 2 and 3, while Groups 2 and 3 did not differ significantly.

TP concentration differed significantly among groups (*p* < 0.01), with higher values observed in Groups 2 and 3 compared with Group 1, whereas Groups 2 and 3 did not differ significantly from each other. UREA concentration also differed among groups (*p* < 0.05), with the highest values observed in Group 3. Pairwise comparisons indicated a significant difference between Groups 1 and 3, whereas other comparisons were not significant.

Enzyme activities associated with liver function and tissue metabolism differed significantly among groups. ALT activity differed among groups (*p* < 0.05), with higher values observed in Group 3 compared with Group 1, while Group 2 did not differ significantly from either Group 1 or Group 3. GGT activity differed significantly among all groups (*p* < 0.001), with values increasing progressively from Group 1 to Group 3. LDH activity also differed significantly among all groups (*p* < 0.001), with the lowest values observed in Group 1 and the highest in Group 3.

GLUC concentration differed significantly among groups (*p* < 0.01), with values increasing from Group 1 to Group 3. Mg concentration differed among groups (*p* < 0.01), with higher values observed in Group 3 compared with Groups 1 and 2, while Groups 1 and 2 did not differ significantly. TRIG concentration differed significantly among groups (*p* < 0.001), with higher values observed in Group 1 compared with Groups 2 and 3, whereas Groups 2 and 3 did not differ significantly.

### 3.3. Univariable Regression Analysis

The results of the univariable linear regression analysis are presented in Table 4. Rumination time was used as the dependent variable, and each milk and blood parameter was analyzed separately as an independent variable. Several variables showed statistically significant associations with rumination time (*p* < 0.05), including electrical milk conductivity, creatinine, magnesium, potassium, and chloride. The regression coefficients indicate the direction of the associations, with positive coefficients for electrical conductivity, creatinine, and potassium, and negative coefficients for magnesium and chloride. Because the regression analysis was based on univariable models, these associations should be interpreted as exploratory and not as independent predictors.

## 4. Discussion

This study combined precision monitoring with biochemical and milk trait analysis to explore the relationship between physiological status and rumination behavior in early-lactation dairy cows. However, because of the observational and cross-sectional design, it is not possible to determine whether rumination time directly influences these parameters or whether it acts as an integrative indicator reflecting the physiological and metabolic status of the cow. In this context, rumination time should be interpreted as a multifactorial indicator influenced by nutritional status, metabolic balance, stage of lactation, and health status rather than as a single causal factor. Cows classified in the low RT group exhibited several differences in milk quality and biochemical parameters compared with cows showing moderate or high RT, suggesting that rumination behavior may reflect underlying physiological variation during early lactation [17].

Lower RT was associated with higher body temperature, SCC, and ECM. Elevated SCC and ECM are commonly used as proxy indicators associated with udder health status and may reflect subclinical changes in the mammary gland. In the present study, cows with clinically evident mastitis were excluded during the clinical examination; therefore, the observed differences in SCC and ECM likely reflect subclinical variation rather than confirmed clinical disease. Previous studies have shown that cows developing mastitis may exhibit behavioral changes, including reduced rumination activity prior to clinical diagnosis [18]. MP percentage was lower in cows with reduced RT compared with cows showing higher rumination activity. This difference may partly reflect production-related effects. Although lower milk protein concentration is sometimes explained by a dilution effect associated with higher MY, it did not differ significantly among rumination groups in the present study. Similar associations between rumination behavior and milk composition have been reported previously, although the direction and magnitude of these relationships may vary depending on production level and metabolic status [19]. Therefore, the lower MP observed in cows with reduced RT may be associated with metabolic or nutritional factors, such as differences in energy balance, feed intake, or nutrient utilization during early lactation, rather than production level alone [17]. However, because feed intake and energy balance were not directly measured in this study, this interpretation should be considered with caution.

Several biochemical indicators differed among rumination groups. TP concentrations were higher in cows with lower RT, while ALB concentrations did not differ significantly among groups. Because ALB typically decreases during systemic inflammatory responses, the absence of differences in ALB suggests that the observed variation in TP may be influenced by factors other than systemic inflammation, such as hydration status or changes in globulin fractions [20,21]. However, because globulin fractions, hydration indicators, and repeated measurements were not assessed in the present study, this interpretation should be considered tentative, and the observed differences in total protein should be interpreted with caution.

Cows with reduced RT also showed higher UREA concentrations and higher activities of GGT and ALT. These findings may be associated with metabolic changes during early lactation. Because ALT has limited diagnostic value in cattle, these enzyme changes should be interpreted cautiously and cannot be used alone to indicate liver disease. Instead, they may reflect metabolic adaptation or increased metabolic load during early lactation.

LDH activity was also higher in cows with reduced RT. LDH is a general indicator of tissue metabolic activity and cellular turnover and has low tissue specificity; therefore, it cannot be used to identify the specific organ or process involved [22]. Consequently, the biological interpretation of increased LDH activity is limited, and the observed differences may reflect general metabolic activity or physiological load rather than a specific pathological process [23].

TRIG concentrations were lower in cows with reduced rumination activity. Reduced circulating TRIG during early lactation may be associated with increased lipid mobilization and metabolic adaptations related to negative energy balance in high-producing dairy cows. These metabolic changes are common during the transition period and may influence feeding behavior and rumination patterns [24].

CREA also showed an association with RT, suggesting that rumination behavior may be linked to metabolic processes related to muscle metabolism, hydration status, or overall physiological activity. However, previous studies have reported variable relationships between rumination activity and CREA concentrations, indicating that this association may depend on physiological context and management conditions [25]. In addition, because the regression analysis was univariable and did not control for potential confounding factors such as days in milk, parity, or MY, this association should be interpreted as exploratory and not necessarily as an independent relationship.

The observed relationship between K concentration and RT may be related to the role of K in rumen function and neuromuscular activity, as adequate potassium availability contributes to ruminal motility and digestive processes. However, similar to CREA, this association should be interpreted with caution because the regression models did not include potential confounding variables, and therefore, the observed relationships may reflect general physiological variation rather than direct effects on rumination behavior [26].

Overall, the findings of this study suggest that RT integrates signals from multiple physiological systems, including metabolic status, milk production, and mineral balance. Monitoring rumination behavior through precision livestock technologies may therefore provide valuable information for the early identification of cows experiencing metabolic or health-related challenges during early lactation. Because cows with clinical disease were excluded, the observed differences likely represent subclinical physiological variation rather than clinically diagnosed disorders.

Several limitations should be considered when interpreting these results. The study was conducted on a single commercial dairy farm, which may limit the generalizability of the findings. In addition, cows were included between 2 and 100 days in milk, and stage of lactation may have acted as a confounding factor influencing metabolic and production parameters. Parity was also not included as a covariate in the statistical models. Blood samples were collected at a single time point, and rumination groups were based on daily rumination time values, which may not reflect longer-term patterns. Furthermore, the regression analysis was based on univariable models and did not control for potential confounding variables; therefore, the observed associations should be interpreted as exploratory. Future studies including multiple farms, longitudinal data, and multivariable statistical models would help to better evaluate the relationship between rumination time and physiological parameters in dairy cows.

## 5. Conclusions

In conclusion, reduced rumination time in early-lactation dairy cows was associated with differences in milk quality and several biochemical parameters, including total protein, urea, selected enzyme activities, triglycerides, and electrolytes. Because cows with clinical disease were excluded from the study, these differences most likely reflect subclinical physiological variation rather than clinically diagnosed disorders. Rumination time is influenced by multiple physiological and management-related factors, including metabolic status, stage of lactation, and production level, and therefore should be interpreted as a multifactorial indicator rather than a single diagnostic parameter. Within the limitations of this observational study, rumination time appears to function as an integrative indicator of overall physiological status rather than a direct causal factor affecting metabolic or milk parameters. The combination of rumination monitoring with milk and biochemical indicators may provide a useful supportive tool for identifying cows that may require closer monitoring during early lactation. However, because the analysis did not control for potential confounding factors such as days in milk and parity, the observed relationships should be interpreted as exploratory rather than independent effects. Further studies using larger populations, longitudinal designs, and multivariable statistical models are needed to better clarify these relationships.

## Figures and Tables

**Table 2:** Milk and blood parameters presented as mean ± SD across rumination time groups (one-way ANOVA with Tukey’s post hoc test).

Parameter	Group 1	Group 2	Group 3	*p*-Value
SCC (log_2_)	3.44 ± 0.71 ^a^	3.58 ± 0.67 ^a^	4.12 ± 0.61 ^b^	*p* < 0.001
T, °C	39.00 ± 0.90 ^a^	39.27 ± 0.95 ^ab^	39.50 ± 0.70 ^b^	*p* = 0.032
ECM	71.00 ± 4.81 ^a^	68.00 ± 4.63 ^a^	70.00 ± 2.96 ^a^	*p* = 0.041
Milk protein (%)	3.39 ± 0.20 ^a^	3.58 ± 0.23 ^ab^	3.66 ± 0.39 ^b^	*p* = 0.018
CRP (log)	2.45 ± 0.29 ^a^	2.08 ± 0.40 ^b^	1.87 ± 0.55 ^b^	*p* = 0.006
GLUC (log)	1.07 ± 0.06 ^a^	1.17 ± 0.16 ^b^	1.25 ± 0.18 ^c^	*p* = 0.002
Mg (log)	0.08 ± 0.15 ^a^	0.02 ± 0.22 ^a^	0.16 ± 0.51 ^b^	*p* = 0.008
TP (log)	4.36 ± 0.13 ^a^	4.16 ± 0.16 ^b^	4.19 ± 0.12 ^b^	*p* = 0.04
Urea	4.53 ± 1.07 ^a^	5.00 ± 1.10 ^ab^	5.32 ± 1.32 ^b^	*p* = 0.021
ALT (log)	2.86 ± 0.40 ^a^	2.96 ± 0.33 ^ab^	3.09 ± 0.36 ^b^	*p* = 0.039
GGT (log)	2.55 ± 0.45 ^a^	2.96 ± 0.32 ^b^	3.07 ± 0.42 ^c^	*p* < 0.001
LDH (log)	6.99 ± 0.40 ^a^	7.36 ± 0.38 ^b^	7.56 ± 0.36 ^c^	*p* < 0.001
TRIG (log)	−0.62 ± 0.70 ^a^	−1.51 ± 0.60 ^b^	−1.83 ± 0.55 ^b^	*p* < 0.001

Means within a row with different superscript letters (a–c) differ significantly between rumination groups based on Tukey’s post hoc test (*p* < 0.05).

**Table 3:** Non-normally distributed milk and blood parameters presented as median across rumination time groups (Kruskal–Wallis test).

Parameter	Group 1	Group 2	Group 3	*p*-Value
MF (%)	4.30 [3.70–4.90]	4.25 [3.60–4.95]	4.10 [3.50–4.80]	*p* = 0.412
Lactose (%)	4.47 [4.37–4.58]	4.51 [4.44–4.60]	4.54 [4.45–4.60]	*p* = 0.538
MY (kg/day)	33.40 [27.85–39.55]	34.65 [24.68–45.65]	31.60 [24.15–50.55]	*p* = 0.287
AST (U/L)	78.40 [71.50–86.70]	77.00 [67.03–88.05]	81.50 [70.50–102.05]	*p* = 0.463
NEFA (mmol/L)	0.03 [0.02–0.12]	0.02 [0.02–0.10]	0.02 [0.02–0.14]	*p* = 0.629
Ca (mmol/L)	2.39 [2.21–2.48]	2.32 [2.22–2.48]	2.37 [2.16–2.45]	*p* = 0.517
Na (mmol/L)	139.0 [136.5–141.5]	139.0 [137.3–141.0]	139.5 [135.3–142.0]	*p* = 0.884
K (mmol/L)	4.30 [4.20–4.70]	4.30 [4.00–4.60]	4.20 [4.10–4.50]	*p* = 0.731
Cl (mmol/L)	95.0 [93.0–98.0]	95.5 [93.3–98.0]	96.5 [94.0–99.0]	*p* = 0.268
Fe (µmol/L)	18.0 [14.0–22.0]	16.5 [12.0–21.0]	18.2 [13.0–23.0]	*p* = 0.452
PHOS (mmol/L)	2.06 [1.75–2.30]	1.97 [1.70–2.20]	2.01 [1.65–2.40]	*p* = 0.603
ALB (g/L)	33.6 [30.0–36.0]	33.5 [30.5–36.5]	33.0 [29.0–36.0]	*p* = 0.748
CREA (µmol/L)	52.1 [48.6–56.9]	51.3 [47.9–55.5]	51.9 [47.6–56.7]	*p* = 0.691

Non-normally distributed milk and blood parameters presented as median and interquartile ranges across rumination time groups. Cows were classified into three groups according to daily rumination time: Group 1 (>527 min/day), Group 2 (412–527 min/day), and Group 3 (<412 min/day). Differences among groups were evaluated using the Kruskal–Wallis test. NEFA—non-esterified fatty acid; AST—aspartate aminotransferase; Ca—calcium; Na—sodium; K—potassium; Cl—chloride; Fe—iron; PHOS—phosphorus; ALB—albumin; CREA—creatinine.

**Table 4:** Univariable linear regression analysis of milk and blood parameters associated with rumination time.

Variable	B	SE	95% CI	*p*-Value
ECM (Lely conductivity score)	80.37	40.78	0.46 to 160.29	*p* < 0.05
CREA (mmol/L)	30.45	10.36	10.16 to 50.75	*p* < 0.01
Mg (mmol/L)	−370.29	112.23	−590.27 to −150.32	*p* < 0.01
K (mmol/L)	900.25	395.33	120.91 to 1670.59	*p* < 0.05
Cl (mmol/L)	−130.48	66.40	−260.62 to −0.34	*p* < 0.05

Dependent variable: rumination time (min/day). Confidence interval (CI), regression coefficient (B). Only variables with statistically significant associations with rumination time are presented.

## Data Availability

The original contributions presented in the study are included in the article; further inquiries can be directed to the corresponding author.

## Abbreviations

ALB	Albumin
ALT	Alanine aminotransferase
AST	Aspartate aminotransferase
ANOVA	One-way analysis of variance
Ca	Calcium
Cl	Chloride
CP	Crude protein
CI	Confidence interval
CREA	Creatinine
CRP	C-reactive protein
DIM	Days in milk
DM	Dry matter
ECM	Electrical conductivity of milk
GGT	Gamma-glutamyl transferase
GLUC	Glucose
LDH	Lactate dehydrogenase
Mg	Magnesium
MY	Milk yield
MP	Milk protein
MF	Milk fat
Na	Sodium
NEFA	Non-esterified fatty acid
PHOS	Phosphorus
RT	Rumination time
SCC	Somatic cell count
SD	Standard deviation
SE	Standard error
T	Milk temperature
TP	Total protein
TRIG	Triglyceride
UREA	Urea
